# Blood-meal analysis of *Culicoides* (Diptera: Ceratopogonidae) reveals a broad host range and new species records for Romania

**DOI:** 10.1186/s13071-020-3938-1

**Published:** 2020-02-17

**Authors:** Alexandru Tomazatos, Hanna Jöst, Jonny Schulze, Marina Spînu, Jonas Schmidt-Chanasit, Daniel Cadar, Renke Lühken

**Affiliations:** 1 WHO Collaborating Centre for Arbovirus and Hemorrhagic Fever Reference and Research, Bernhard Nocht Institute for Tropical Medicine, Hamburg, Germany; 2 University of Agricultural Sciences and Veterinary Medicine, Cluj-Napoca, Romania; 3 Faculty of Mathematics, Informatics and Natural Sciences, Universität Hamburg, Hamburg, Germany

**Keywords:** *Culicoides*, Barcoding, Host-feeding patterns, Danube delta, Romania

## Abstract

**Background:**

*Culicoides* biting midges are potential vectors of different pathogens. However, especially for eastern Europe, there is a lack of knowledge on the host-feeding patterns of this vector group. Therefore, this study aimed to identify *Culicoides* spp. and their vertebrate hosts collected in a wetland ecosystem.

**Methods:**

*Culicoides* spp. were collected weekly from May to August 2017, using Biogents traps with UV light at four sites in the Danube Delta Biosphere Reserve, Romania. Vectors and hosts were identified with a DNA barcoding approach. The mitochondrial cytochrome *c* oxidase subunit 1 was used to identify *Culicoides* spp., while vertebrate hosts were determined targeting cytochrome *b* or *16S* rRNA gene fragments. A maximum likelihood phylogenetic tree was constructed to verify the biting midge identity against other conspecific Palaearctic *Culicoides* species. A set of unfed midges was used for morphological confirmation of species identification using slide-mounted wings.

**Results:**

Barcoding allowed the species identification and detection of corresponding hosts for 1040 (82.3%) of the 1264 analysed specimens. Eight *Culicoides* spp. were identified with *Culicoides griseidorsum*, *Culicoides puncticollis* and *Culicoides submaritimus* as new species records for Romania. For 39 specimens no similar sequences were found in GenBank. This group of unknown *Culicoides* showed a divergence of 15.6–16.3% from the closest identified species and clustered in a monophyletic clade, i.e. a novel species or a species without reference sequences in molecular libraries. For all *Culicoides* spp., nine mammalian and 24 avian species were detected as hosts. With the exception of *C. riethi* (*n* = 12), at least one avian host was detected for all *Culicoides* spp., but this host group only dominated for *Culicoides kibunensis* and the unknown *Culicoides* sp.. The most common host group were mammals (*n* = 993, 87.6% of all identified blood sources) dominated by cattle (*n* = 817, 70.6%).

**Conclusions:**

Most *Culicoides* spp. showed a broad host-feeding pattern making them potential bridge vectors. At the same time, new records of biting midge species for Romania, as well as a potentially unknown *Culicoides* species, highlight the lack of knowledge regarding the biting midge species and their genetic diversity in eastern Europe.
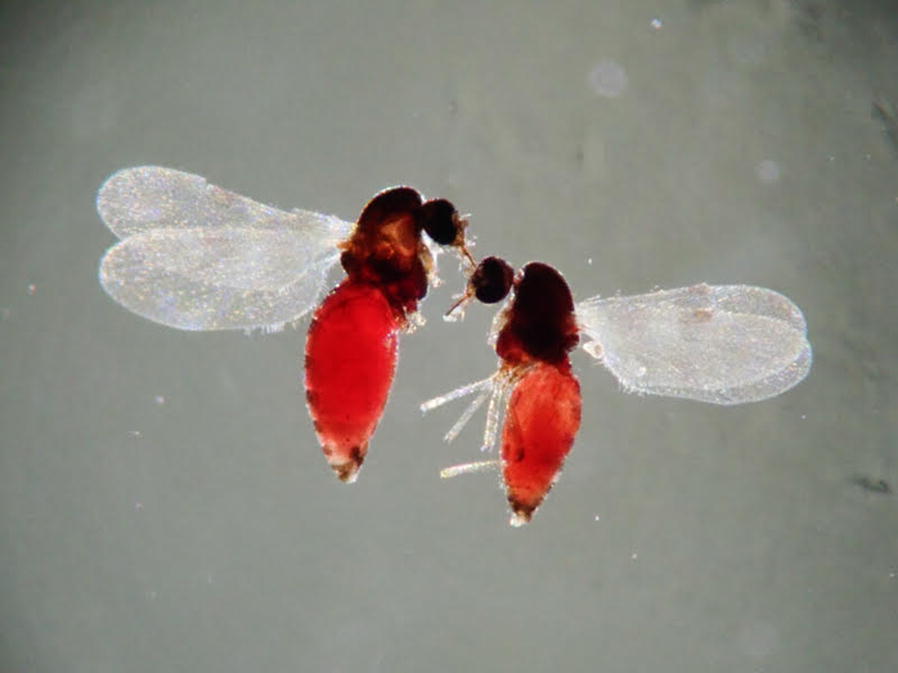

## Background

Biting midges of the genus *Culicoides* Latreille, 1809 (Diptera: Ceratopogonidae) are vectors of a variety of pathogens. These include protozoans [1–3], filarial worms [4] and numerous viruses [5]. Their relevance as vectors is primarily related to veterinary health, though outbreaks of the *Culicoides-*borne Oropouche virus in humans regularly occur in the Neotropics [6]. In Europe, several biting midge species are able to transmit bluetongue virus (BTV), African horse sickness virus and Schmallenberg virus (SBV) [7]. These viruses are responsible for outbreaks of non-contagious diseases in ruminants, causing huge economic losses, e.g. due to restrictions on animal trade [8].

The expansion of BTV from the Mediterranean basin to central Europe up to Scandinavia [9–11] prompted studies on *Culicoides* taxonomy [12–14], ecology [15–17] and vector competence [18–20]. In contrast, only few studies focused on the *Culicoides* fauna in southeastern Europe. Severe BTV outbreaks were observed between 2014 and 2015 in the Balkan Peninsula [21, 22]. In Romania, BTV was confirmed for the first time in 2014 [23]. The most comprehensive studies on the *Culicoides* fauna conducted in Romania date back to the end of the 20th century [24, 25]. More recent studies of the *Culicoides* fauna in Romania only focused on the known vectors of BTV. Thus, with the exception of *C. imicola* Kieffer, 1913 or *C. nubeculosus* (Meigen 1830) [26, 27], biting midges were recorded as species groups considered the most important vectors of BTV/SBV, i.e. *C. obsoletus* group and *C. pulicaris* group, or as “other *Culicoides*” [28, 29]. Currently, species-specific information on the distribution of other *Culicoides* taxa in Romania is missing.

The identification of blood sources from engorged vectors is a useful method to understand vector-host interactions and the ecology of associated pathogens [30, 31]. The host-feeding patterns of *Culicoides* have received much less attention compared to other vector groups (e.g. mosquitoes and ticks) [32, 33]. In Europe, most of the vertebrate hosts identified from engorged biting midges are ruminants [34–36]. However, other mammalian species such as humans and pigs can also be frequent [37–39]. In comparison, avian hosts are generally a more diverse, but less frequent group compared to mammals [34, 37, 38, 40]. Information about hosts of *Culicoides* species from eastern Europe was obtained by recent efforts undertaken in natural areas of Bulgaria [41] and Serbia [42]. In Serbia, blood-meal analysis predominantly detected ruminant hosts, whereas in Bulgaria, a large diversity of avian hosts was recorded for ornithophilic biting midges. To the best of our knowledge, such studies do not exist for Romania. Therefore, the aim of this study was to investigate the host-feeding patterns of *Culicoides* species collected from four sampling sites in the Danube Delta Biosphere Reserve (DDBR).

## Methods

### Trapping methods and study sites

**Fig. 1:**
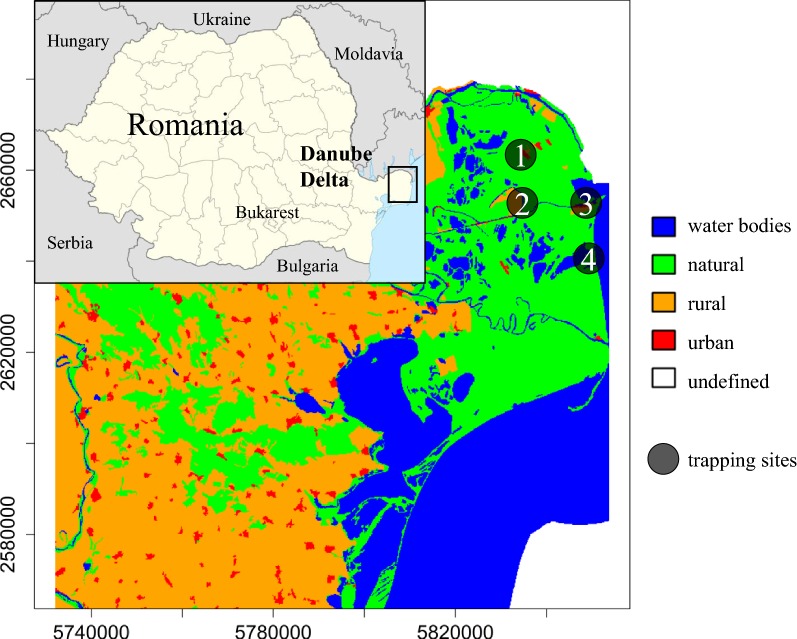
Sampling sites (1: Letea; 2: Dunărea Veche; 3: Sulina; 4: Lake Roșuleț) of *Culicoides* in the Danube Delta Biosphere Reserve (Romania) during the sampling period in 2017. Landcover variables are aggregated land cover data (Corine Land Cover (CLC) 2018 raster data, https://land.copernicus.eu/). CLC codes: water bodies, 511–523; natural, 311–423; rural, 211–244; urban, 111–142

Biting midges were collected at four sites in the DDBR as part of a pilot longitudinal arbovirus surveillance programme [43] (Fig. 1, Additional file 1: Text S1). The trapping site Letea is characterized by a semi-open enclosure for cattle and goats built of wood, reeds and rushes, located a short distance from a small canal and almost 1 km from a deciduous forest. In Sulina, the sampling site was a covered cow stable with two or three animals kept at night with a stagnant water body (canal) and a large dung heap in close proximity. The local host communities of both anthropogenic sites (Letea and Sulina) are predominantly characterized by cattle, horse, cat, poultry and humans accompanied by dogs. In contrast, the site at Dunărea Veche lays at the confluence of two branches of the Danube and adjacent small canals; a large crop field is bordered by these waters. The site Lake Roșuleț is an old fishery surrounded by a shallow, stagnant canal and rows of trees isolating the area from the surrounding marshland. Only few humans (farmers and fishermen) with dogs and cats are present in Dunărea Veche and Lake Roșuleț. The host community of both sites is predominantly characterized by a high diversity of wild mammals and birds.

Between May and August 2017, one Biogents Sentinel trap (BG trap; Biogents, Regensburg, Germany (http://www.biogents.com/)) equipped with an ultraviolet lamp was operated at each site for one night per week resulting in a total of 60 trap nights. The climate of the study area is continental with an annual mean temperature of 11 ℃ (− 1 ℃ in January and 22 ℃ in July) and around 350 mm of mean precipitation per year. Sampling in the present study was conducted during a hot and dry summer. A mean temperature of 21 ℃ and mean precipitation under 30 mm was recorded in the Danube Delta between May and August 2017 (http://www.meteoromania.ro/clima/monitorizare-climatica/).

### Sample processing

Insects were frozen, shipped on dry ice and stored at − 80 ℃ in the laboratory. Due to the large amount of non-engorged and engorged *Culicoides*, only a random subsample of 1264 engorged specimens from all four sampling sites and every month of collection were selected. During the progress of sequencing, a dominance of cattle was observed for the sites Sulina and Letea. Therefore, we focused specifically on the engorged *Culicoides* from the sites Dunărea Veche and Lake Roșuleț, where a wider range of wildlife host blood meals were likely to be detected. Dry, frozen storage was preferred over ethanol storage to allow virus isolation and characterization at a later time. Biting midges were separated by engorged status and wing patterns under a stereomicroscope (Olympus ZSX12, Tokyo, Japan). In addition, a small set of unfed specimens (*n* = 37) from each sampling site (Sulina, *n* = 10; Letea, *n* = 9; Dunărea Veche, *n* = 10; Lake Roșuleț, *n* = 8) were used for morphological identification, which were selected as morphologically representative for the different *Culicoides* species in the samples. Wings were mounted on slides in Euparal (Carl Roth, Karlsruhe, Germany) and species identified by morphology using the key of Mathieu et al. [14].

For DNA extraction, each specimen was placed into an individual sterile 2 ml tube (Eppendorf, Hamburg, Germany) with 5–9 zirconium beads (1 mm, Carl Roth) and 200 μl of Dulbecco’s modified Eagle’s medium (Sigma-Aldrich, St. Louis, MO, USA) with 100 μg/ml streptomycin (PAN-Biotech, Aidenbach, Germany) and 2.5 μg/ml amphotericin B (PAN-Biotech). The samples were homogenised with a TissueLyser II (Qiagen, Hilden, Germany) twice for 3 min at 30 Hertz. The suspension was clarified by centrifugation at 8000× *rpm* for 2 min at 4 ℃. Total nucleic acid was extracted from 100 μl of supernatant, using the MagMAX™RNA/DNA Pathogen Kit with a KingFisher™ Flex Magnetic Particle Processor (Thermo Fisher Scientific, Waltham, MA, USA).

### Molecular identification of biting midges

A 658-bp fragment of the mitochondrial cytochrome *c* oxidase subunit 1 gene (*cox*1) was amplified PCR, using the primers HCO2198 and LCO1490 [44]. One microliter template was added to a 10 μl reaction mix, containing 6.6 μl of Hotstar Taq Master Mix (Qiagen), 2.2 μl of molecular grade water (included in the Master Mix kit) and 0.6 μl of each 10 μM primer. The following cycling program was used: initial denaturation at 95 ℃ for 15 min, followed by 40 cycles of 30 s denaturation at 94 ℃, 45 s annealing at 40 ℃ and 1 min extension at 72 ℃, and final extension step for 10 min at 72 ℃. Each PCR run included DNA of *Culex quinquefasciatus* Say, 1823 (positive control) and ultrapure water (negative control). All amplicons were visualised on 2% agarose gels and PCR products sequenced with LGC Genomics (Berlin, Germany).

### Molecular identification of *Culicoides* hosts

Hosts were identified using two PCR protocols targeting the cytochrome *b* (*cytb*) and *16S* rRNA gene fragment [45–47]. Both protocols were described in detail in a previous study by Börstler et al. [32]. If the amplification with the first pair of primers failed [45, 46], another PCR was applied using the second pair of primers [47]. The same applied to potential mixed blood meals as indicated by double peaks at different positions in the sequence electropherograms. These samples were also analysed with both PCRs. As observed in our previous studies [32, 33], the PCR targeting the *cytb* gene fragment generally has a higher amplification rate for mammals, and the PCR targeting the *16S* rRNA gene fragment a higher amplification rate for birds. We used the DNA of a mammal (African green monkey, *Chlorocebus sabaeus* (L.)) and a bird (European blackbird, *Turdus merula* L.) as positive controls. The negative control was ultrapure water, which was included in each PCR run. These amplicons were also visualised and sequenced as described above.

### Data analysis

Sequences were visualised and edited with Geneious version 9.1.7 (Biomatters, Auckland, New Zealand). The resulting sequences were submitted for species identification using the basic alignment search tool (BLAST) in the GenBank DNA sequence database (https://blast.ncbi.nlm.nih.gov/) and the Barcode of Life Database [48]. In order to rule out potential contamination, samples indicating human host DNA were repeated separately in an individual PCR reaction. Identity values for the *Culicoides* and host species generally ranged between 98 and 100%. Sequences with lower identity values were repeated. One exception was the newly described haplotype of *C. punctatus* (Meigen, 1804), which showed identity values between 96 and 97%. In addition, information on the fauna of the DDBR were used to interpret the sequences. For example, domestic pig has become quite a rarity in the study area (Additional file 1: Text S1). Therefore, these sequences were classified as wild boar, which is a common wild mammal in the DDBR.

To assess the phylogenetic relationship of *Culicoides* identified in the DDBR with other previously reported species in the Palaearctic, a maximum likelihood tree was constructed with MEGAX [49] with additional conspecific and outgroup sequences (*Forcipomyia* spp. and *Cx. quinquefasciatus*) from GenBank (Additional file 2: Table 1). The HKY + G model was identified as the best-fit model of nucleotide substitution by Jmodeltest 2.1.10 [50] based on calculations of Bayesian and Akaikeʼs information criteria. Robustness of nodes was assessed by 1000 bootstrap replicates. The *Culicoides* spp. sequences generated in this study were deposited in the GenBank database under the accession numbers MN274523-274532 and MN340302-340312.

**Table 1:** Frequency of detected hosts per *Culicoides* spp. with corresponding percentage collected in the Danube Delta Biosphere Reserve (Romania) during 2017

Host	*C. griseidorsum n* (%)	*C. kibunensis n* (%)	*C. punctatus n* (%)	*C. punctatus* P *n* (%)	*C. riethi n* (%)	*C. subfasciipennis/C. pallidicornis n* (%)	*C. submaritimus n* (%)	Unknown *Culicoides* sp. *n* (%)	Host information without *Culicoides* identification *n* (%)	Total *n* (%)
Mammals (*n *= 1064, 92%)										
*Bos taurus* L.	170 (63.9)	4 (5.2)	207 (85.9)	163 (81.1)	8 (80.0)	188 (83.2)	1 (11.1)	2 (11.1)	74 (68.5)	817 (70.7)
*Bubalus bubalis* (Kerr)			1 (0.4)			1 (0.4)				2 (0.2)
*Canis lupus familiaris* (L.)		4 (5.2)				3 (1.3)				7 (0.6)
*Capra hircus* L.	46 (17.3)	1 (1.3)	3 (1.2)	1 (0.5)	1 (10.0)	1 (0.4)				53 (4.6)
*Capreolus capreolus* (L.)		1 (1.3)								1 (0.1)
*Equus caballus* L.	15 (5.6)		9 (3.7)	3 (1.5)	1 (10.0)	4 (1.8)		1 (5.6)	5 (4.6)	38 (3.3)
*Felis catus* L.		1 (1.3)				1 (0.4)				2 (0.2)
*Homo sapiens* L.	3 (1.1)	10 (13.0)	5 (2.1)	4 (2.0)		4 (1.8)	5 (55.6)	2 (11.1)	10 (9.3)	43 (3.7)
*Sus scrofa* L.	28 (10.5)	3 (3.9)	16 (6.6)	29 (14.4)		22 (9.7)			3 (2.8)	101 (8.7)
Birds (*n* = 92, 8%)										
*Acrocephalus arundinaceus* (L.)		1 (1.3)				1 (0.4)				2 (0.2)
*Acrocephalus scirpaceus* (Hermann)		13 (16.9)								13 (1.1)
*Ardea cinerea* L.		1 (1.3)								1 (0.1)
*Ardea purpurea* L.		6 (7.8)							1 (0.9)	7 (0.6)
*Columba palumbus* L.		1 (1.3)						1 (5.6)		2 (0.2)
*Coracias garrulus* L.		2 (2.6)						3 (16.7)		5 (0.4)
*Corvus corone* L.	1 (0.4)	6 (7.8)					3 (33.3)		6 (5.6)	16 (1.4)
*Cyanistes caeruleus* (L.)		4 (5.2)		1 (0.5)				1 (5.6)		6 (0.5)
*Emberiza schoeniclus* (L.)									1 (0.9)	1 (0.1)
*Falco tinnunculus* L.								1 (5.6)	1 (0.9)	2 (0.2)
*Gallinula chloropus* (L.)		3 (3.9)								3 (0.3)
*Gallus gallus* (Gmelin)	2 (0.8)	2 (2.6)						2 (11.1)		6 (0.5)
*Hirundo rustica* L.								2 (11.1)		2 (0.2)
*Meleagris gallopovo* L.									1 (0.9)	1 (0.1)
*Motacilla alba* L.		1 (1.3)								1 (0.1)
*Nycticorax nycticorax* (L.)		1 (1.3)				1 (0.4)		1 (5.6)		3 (0.3)
*Parus major* L.		1 (1.3)							3 (2.8)	4 (0.3)
*Passer montanus* (L.)		1 (1.3)								1 (0.1)
*Phalacrocorax carbo* (L.)									2 (1.9)	2 (0.2)
*Streptopelia decaocto* (Frivaldszky)								2 (11.1)		2 (0.2)
*Strix aluco* L.		1 (1.3)								1 (0.1)
*Sylvia borin* (Boddaert)	1 (0.4)	4 (5.2)								5 (0.4)
*Tito alba* (Scopoli)		1 (1.3)								1 (0.1)
*Asio otus* (L.)		2 (2.6)								2 (0.2)
*Tito alba*/*Asio otus*		2 (2.6)							1 (0.9)	3 (0.3)
*Culicoides* specimens without host identification	11	26	7	6	2	19	1	21		
Total biting midge specimens	276^a^	102^b^	248	207	12	242^c^	8^d^	39^e^		

^a^Including one mixed blood meal: *Bos taurus* + *Gallus gallus*

^b^Including one mixed blood meal: *Sus scrofa* + *Homo sapiens*

^c^Including three mixed blood meals: *Bos taurus* + *Canis lupus familiaris*; *Sus scrofa* + *Acrocephalus arundinaceus*; *Bos taurus* + *Nycticorax nycticorax*

^d^Including two mixed blood meals: *Corvus corone* + *Homo sapiens*

^e^Including one mixed blood meal: *Equus caballus* + *Hirundo rustica*

## Results

### Molecular identification of biting midges

**Fig. 2:**
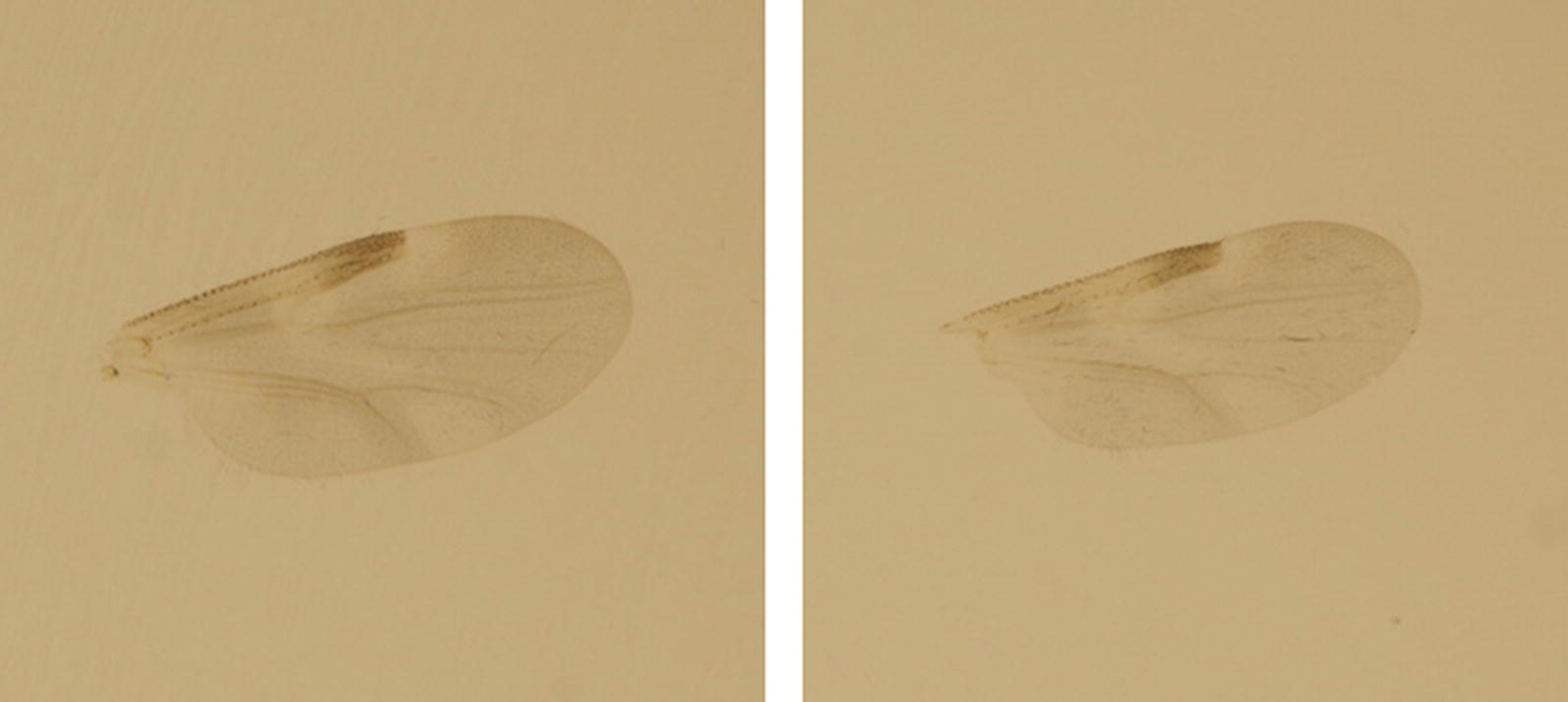
Two wing pictures for the unknown *Culicoides* species collected in the Danube Delta Biosphere Reserve (Romania) during 2017

Sequencing a fragment of the *cox*1 gene allowed the molecular identification of 1134 (89.7%) of the analysed 1264 engorged *Culicoides* (Table 1). Five species were identified for engorged biting midges: *C. griseidorsum* Kieffer, 1918; *C. kibunensis* Tokunaga, 1937; *C. punctatus*; *C. riethi* Kieffer 1914; and *C. submaritimus* Tokunaga & Murachi, 1959. *Culicoides subfasciipennis* Kieffer, 1919/*C. pallidicornis* Tokunaga & Murachi, 1959 were not differentiated to the species level. Furthermore, 39 sequences (3.1% of the analysed specimens) could not be identified to species level by comparison with other *Culicoides* sequences available on GenBank. The sequences of these specimens had a high similarity indicative of belonging to the same species and represent the seventh taxon hereafter referred to as “unknown *Culicoides*”. The eighth taxon detected was *C. puncticollis* (Becker, 1903), only present in the non-engorged fed biting midges selected for morphological identification. Four of the seven detected engorged species were confirmed by morphology: *C. griseidorsum*; *C. kibunensis*; *C. riethi*; and *C. punctatus.* In contrast, engorged *C. submaritimus* and *C. subfasciipennis*/*C. pallidicornis* were identified solely by barcoding and were not found in the small set of unfed specimens. *Culicoides puncticollis* was identified by morphology and *cox*1 barcoding, but only from the same subset of 37 unfed specimens (Additional file 3: Figure S1). As the *cox*1 sequences are not suitable to differentiate between *C. subfasciipennis* and *C. pallidicornis* [51, 52], these specimens were classified as *C. subfasciipennis*/*C. pallidicornis*. The unknown *Culicoides* species had similar wing patterns to *C. kibunensis* (Fig. 2).

**Fig. 3:**
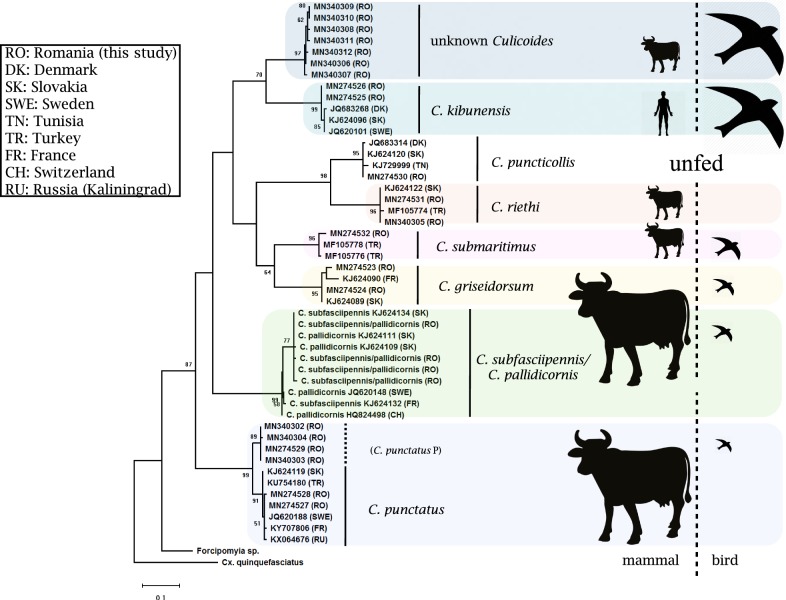
Maximum likelihood phylogenetic tree of *cox*1 sequences for *Culicoides* species collected in the Danube Delta Biosphere Reserve (Romania) during 2017. Silhouettes indicate observed host-feeding patterns regarding the relative frequencies of mammalian and avian hosts. The tree was inferred using an HKY + G model (1000 bootstrap replicates) and rooted with *Forcipomyia* sp. and *Culex quinquefasciatus*. Branch support values of ≥ 50% are displayed and GenBank accession numbers of sequences shown on the branch tips

In order to perform a identity verification of the generated *Culicoides cox*1 sequences, we constructed a maximum likelihood phylogenetic tree including conspecific *Culicoides* and outgroup sequences (Fig. 3). A distinct haplotype of *C. punctatus* (designated as *C. punctatus* P) was identified in almost half (*n* = 207, 45.5%) of the 454 *C. punctatus* specimens analysed. These clustered within a separate monophyletic clade showing a genetic distance of approximately 4% to *C. punctatus* (Fig. 3). For the unknown *Culicoides* we could not find any similar sequences in the databases. This group of specimens showed a divergence of 15.6–16.3% from the closest identified *Culicoides* species (data not shown). The sequences of these specimens had a high similarity with each other and clustered with *C. kibunensis* in a monophyletic clade (Fig. 3).

*Culicoides punctatus* (*n* = 455, 36.0% of all analysed specimens), *C. griseidorsum* (*n* = 276, 21.8%), *C. subfasciipennis*/*C. pallidicornis* (*n* = 242, 19.1%) and *C. kibunensis* (*n* = 102, 8.1%) were the most frequent taxa identified (Table 1, Additional file 4: Table S2). *Culicoides riethi* (*n* = 12, 0.95%) was found in the traps set near livestock in the sites Sulina and Letea, while *Culicoides submaritimus* (*n* = 8, 0.63%) was only found for Dunărea Veche and Lake Roșuleț, respectively.

### Molecular identification of *Culicoides* hosts

Overlapping the two sets of sequences obtained for *Culicoides* identification and their hosts, information was available for 1040 (91.7%) of the 1134 molecular identified biting midges (Table 1). Blood-meal identification was not possible for 93 specimens due to failed PCR amplification. In addition, eight mixed blood meals were detected. With the exception of *C. punctatus* (*n* = 455) and *C. riethi* (*n* = 12), mixed blood meals where found for engorged specimens of all five *Culicoides* spp. Two *Culicoides* specimens contained blood from two mammalian hosts, while the other six specimens had mixed blood meals from a bird and a mammal.

A total of 33 vertebrate species were identified including nine species of mammals (27.3%) and 24 species of birds (72.7%) (Table 1). Mammals dominated the host spectrum (*n* = 1064, 92.0% of all 1156 identified blood sources). Cattle (*Bos taurus*) was the most abundant species (*n* = 817, 70.7%), followed by wild boar (*n* = 101, 8.7%). Other mammalian hosts were each found at a rate below 5%. Birds amounted to 8% of all the identified hosts with the Eurasian reed warbler (*Acrocephalus scirpaceus*; *n* = 13, 1.12%) and the carrion crow (*Corvus corone*; *n* = 16, 1.38%) as most frequent.

With the exception of *C. riethi* (*n* = 12), at least one avian host was detected for all *Culicoides* spp. Birds dominated the blood-meal sources of *C. kibunensis* and the unknown *Culicoides* sp. (68.8% and 72.2% of the detected hosts, respectively) (Table 1). *Culicoides kibunensis* had the highest diversity of hosts, with seven (77.8%) of the nine mammalian hosts and 18 (75%) of 24 species of avian hosts. Furthermore, humans were the most frequent mammalian host for this species (*n* = 10, 13.0% of all identified hosts). In contrast, the three most frequent *Culicoides* spp. (*C. griseidorsum*, *C. punctatus* and *C. subfasciipennis*/*C. pallidicornis*) showed high proportions of cattle (between 63.9 and 85.9% of all identified blood sources per taxon). The second most frequent hosts were goat (*Capra hircus*) for *C. griseidorsum* (17.3%) and wild boar for *C. punctatus* (6.6%), *C. punctatus* P (14.4%) and *C. subfasciipennis*/*C. pallidicornis* (9.7%) (Table 1). No differences were observed between *C. punctatus* and its distinct haplotype *C. punctatus* P. Furthermore, for *C. submaritimus* (*n* = 8) only blood meals from humans (*n* = 5), carrion crows (*n* = 3) and cattle (*n* = 1) were detected.

## Discussion

The relevance of *Culicoides* spp. as important vectors of pathogens is well known. Thus, information about their diversity and host-feeding patterns is crucial to understand parasite-host interactions and the ecology of associated pathogens [30]. DNA barcoding is an important tool in biodiversity studies [53–57]. Thereby, barcoding also helped to identify cryptic and new *Culicoides* species [58–60]. In this study, successful sequencing of 1040 engorged insects demonstrated that barcoding is a useful tool for both, *Culicoides* and host identification. However, it must be considered that the different genetic markers can have pitfalls and do not necessarily reflect morphological differences [56, 61], i.e. using a single marker might be insufficient for an accurate identification of species.

A total of seven *Culicoides* species-level taxa were detected for the four sites in the DDBR. In the phylogenetic tree, specimens of the same taxon clustered in well-supported terminal clades. The only exception was *C. subfasciipennis*/*C. pallidicornis*. The separation between these two species is based on a variable light spot on the wing’s anal cell of *C. subfasciipennis* [14]. However, the analysis indicated no sequence differences of the *cox*1 gene. The discriminatory characters on the wing might be unreliable and further studies are required to clarify the status of both species [51, 52].

*Culicoides griseidorsum*, *C. puncticollis* and *C. submaritimus* were recorded for the first time in Romania, increasing the number of known *Culicoides* species for the country to 49 species [25]. *Culicoides submaritimus* has been considered a synonym of *C. maritimus* Tokunaga, 1940 by some authors [62, 63], while recent studies treated *C. submaritimus* as a distinct species [14, 64]. In the present study, *C. submaritimus* was identified by its similarity with *cox*1 sequences from Turkey, which are the only sequences available on GenBank for this species, while no *cox*1 sequences were available for *C. maritimus*. Neither *C. submaritimus*, nor *C. maritimus* are included in the inventory of *Culicoides* biting midges of Romania [25], although more recent studies include the country in the distribution of *C. maritimus* [14, 65].

The observed genetic variation for the analysed *C. punctatus* in two distinct clades is within intraspecific boundaries [59]. Such sibling species may vary in their vectorial capacity [66], e.g. vector competence or host-feeding patterns of members in the *Anopheles gambiae* complex. However, we did not detect differences in the host-feeding patterns between either taxa. Furthermore, the specimens clustering within the clade designated as “unknown *Culicoides*” showed genetic distances of 15.6–16.3% from the closest described species. These distances are similar to those observed between the other *Culicoides* species in our study. Comparable distances were found in other *Culicoides* spp. [67, 68] or mosquitoes [69], indicating that these specimens belong to a separate new species or a species without reference sequences in molecular libraries.

The overall host spectrum covered species expected for the DDBR, including livestock species like buffalo (*Bubalis bubalis*). Therefore, most of the analysed *Culicoides* spp. had a broad host-feeding range. Only mammalian hosts were detected for *C. riethi*, but the small sample size of only 12 engorged specimens does not allow an accurate conclusion on the species’ host-feeding pattern. Both, mammalian and avian hosts were detected for all other biting midge taxa to various extents. The broad host choice matches previous studies, which find similar results for different *Culicoides* spp. [70, 71]. Humans and carrion crow were the only hosts of *C. submaritimus* (*n* = 8). Cattle, wild boar or goat dominated the hosts of the three most frequent *Culicoides* taxa (*C. punctatus*, *C. subfasciipennis*/*C. pallidicornis* and *C. griseidorsum*). The high frequency of cattle probably relates to the large number of free-roaming cattle available in the DDBR and their large body mass [72]. However, as observed before [41, 67, 73, 74], despite this distinct dominance of mammalian hosts, different avian hosts were detected for the three *Culicoides* taxa.

*Culicoides kibunensis* is considered predominantly ornithophilic [37, 38, 75, 76]. With 18 species of birds and seven species of mammals, this vector of avian malaria [37, 38] showed the highest overall host diversity. The wide range of bird species is not surprising, considering the diversity of this vertebrate group in the DDBR. Nevertheless, the observed generalist host-feeding pattern including humans match previous studies [34, 37, 38]. Interestingly, the unknown *Culicoides* species showed a similar host-feeding pattern as *C. kibunensis*, with which it formed a monophyletic clade in the phylogenetic tree. These observations support the hypothesis of a positive correlation between biting midge phylogenetic relatedness and their feeding behaviour [40, 77]. In contrast, other studies speculated that such similarities in host-feeding patterns are not necessarily driven by phylogenetic relatedness, but might be the result of other factors (e.g. body size-driven host choice due to larger emissions of CO_2_ or volatile compounds) [71].

Host availability probably has a significant impact on the observed host-feeding patterns of *Culicoides* spp. Although no quantitative information on the host community is available, the prevalence of humans and domestic animals at Dunărea Veche and Lake Roșuleț is known. Humans, dogs and cats had relative low abundance at both sites compared to birds or free-ranging cattle and horses. Nevertheless, humans, dogs or cats were detected as hosts for all analysed *Culicoides* species. Thus, caution regarding the distribution of biting midges and the potential host has to be considered when interpreting host-feeding patterns of *Culicoides*. For example, a high proportion of *C. griseidorsum* were found to have fed on goats, but this host was widely available at Letea, where most of this species were collected (Additional file 4: Table S2, Additional file 5: Table S3).

Information on the host-feeding patterns can be also used to estimate dispersal distances of *Culicoides* spp. [77]. Biting midges from the sampling site Dunărea Veche were engorged with blood from buffalo and goat. These hosts are only available in the nearest village more than 4 km from the trapping site, which is in the range of a previous study on *Culicoides* [78]. Maximum dispersal distances of more than 3 km over one night were recorded regularly. Winds over the delta’s flat landscape might favour passive dispersal [79–82]. Thereby, besides active midge movement, wind dispersal is considered an important mode of long-distance dispersal for *Culicoides*-borne pathogens [83–85].

## Conclusions

The broad host range of different mammalian and avian species indicates that most of the analysed *Culicoides* species in the DDBR are potential bridge vectors. However, the actual vector competence of these species is largely unknown. Of the dominant *Culicoides* species analysed, *C. punctatus* was previously indicated as a potential vector of BTV and SBV [86, 87]. Free roaming cattle, the most abundant and most frequently detected hosts in the region, could have an important role in amplification and spread of pathogens between wild ruminants and livestock [88]. At the same time, the new records of biting midge taxa for the country presented here and the detection of a potentially unknown *Culicoides* taxon highlight the lack of knowledge regarding the biting midge species and their genetic diversity in Europe.

## Supplementary information

**Additional file 1: Text S1.** Description of the sampling sites with information on vegetation, surrounding environment and available hosts.

**Additional file 2: Table S1.** Accession numbers of *Culicoides* spp., *Forcipomyia* spp. and *Culex quinquefasciatus* used for phylogenetic analysis.

**Additional file 3: Figure S1.** Wing patterns for *C. punctatus*, *C. punctatus* P, *C. kibunensis*, *C. puncticollis*, *C. riethi* and *C. griseidorsum* collected in this study.

**Additional file 4: Table S2.** Overview of the *Culicoides* species per sampling site.

**Additional file 5: Table S3.** Overview of the frequency of each molecularly identified *Culicoides* host species per sampling site.

## Data Availability

The data supporting the conclusions of this article are included within the article and its additional files.

## Abbreviations

DDBR: Danube delta biosphere reserve
DNA: Deoxyribonucleic acid
BTV: Bluetongue virus
SBV: Schmallenberg virus
BG trap: Biogents sentinel trap
PCR: Polymerase chain reaction
