# Supplementary material for: Blood-meal analysis of Culicoides (Diptera: Ceratopogonidae) reveals a broad host range and new species records for Romania
Source: Parasit Vectors. 2020 Feb 17;13:79. doi: 10.1186/s13071-020-3938-1 (PMC7027113; doi:10.1186/s13071-020-3938-1)
Supplement: Supplementary file 1 — Additional file 1: Text S1. Description of the sampling sites with information on vegetation, surrounding environment and available hosts. [file 13071_2020_3938_MOESM1_ESM.docx]

**site 1: Letea - 45°17′13.37” N; 29°30′57.41” E**

Semi-open enclosure for cattle and goats built of wood, reeds and rushes, located ca. 120 meter from a small canal and almost 1 km from a deciduous forest. A small patch of swamp with thick rushes in the vicinity.

**note on local host diversity:** cattle, horse, human, poultry, goat, dog, fox, rabbit, roe deer, jackal, wild boar, high diversity of birds

**site 2: Dunărea Veche - 45°10′27.16” N; 29°28′43.74” E**

The confluence of two Danube branches and adjacent small canals. A large crop field is bordered by these waters. Traps were set between a small canal and the Danube river and in a garden, between the river and marshes.

**note on local host diversity:** several farmers with 6-8 dogs, several cats, wild boar, jackal, rodents, fox, rabbit, cattle, horse, very high diversity of birds

**site 3: Sulina - 45°09′25.09” N; 29°39′14.96” E**

A covered cow stable with two or three animals kept at night. A stagnant water body (canal) with thick vegetation (*Phragmites australis* and *Typha angustifolia*) and a large dung heap between water and the stable. Numerous structures and animal enclosures made of wood, reeds and rushes. The sites are sheltered from the winds by false acacia (*Robinia pseudoacacia*) and shrubs.

**note on local host diversity:** cattle, horse, dog, cat, human, poultry, rodents, fox, rabbit, high diversity of birds

**site 4: Lake Roșuleț - 45°03′51.19” N; 29°37′52.56” E**

An old fishery bordered by a shallow, stagnant canal and one or two rows of willow (*Salix alba*) and ash (*Fraxinus pallisae*), which isolate the area from the surrounding marshland. Located between a complex of lakes, channels, swamps and the Black Sea.

**note on local host diversity:** 1-6 fishermen with three dogs, ca. 15 cats, jackal, rodents, wild boar, cattle, horse, very high diversity of birds
