# Supplementary figures and images for: Blood-meal analysis of Culicoides (Diptera: Ceratopogonidae) reveals a broad host range and new species records for Romania
Source: Parasit Vectors. 2020 Feb 17;13:79. doi: 10.1186/s13071-020-3938-1 (PMC7027113; doi:10.1186/s13071-020-3938-1)

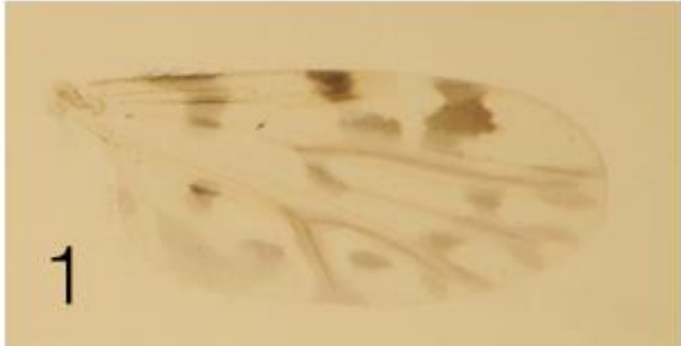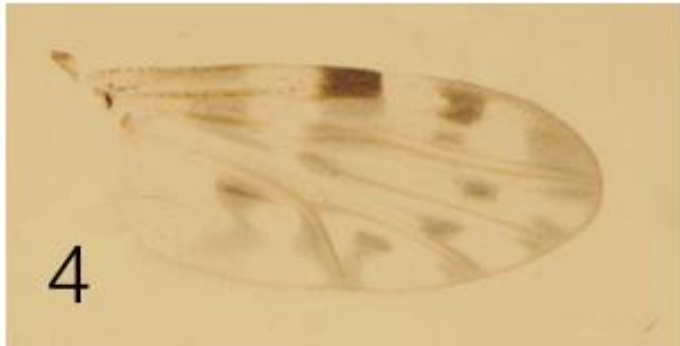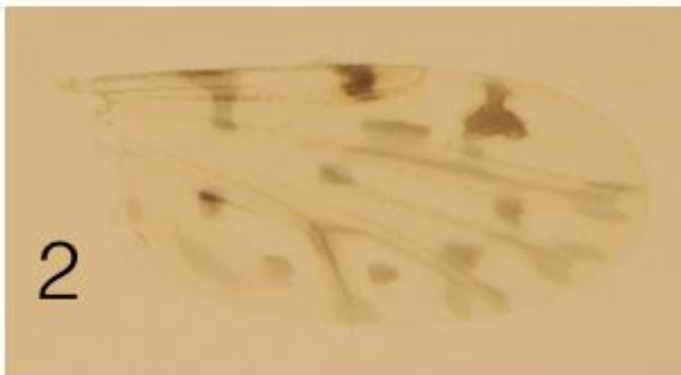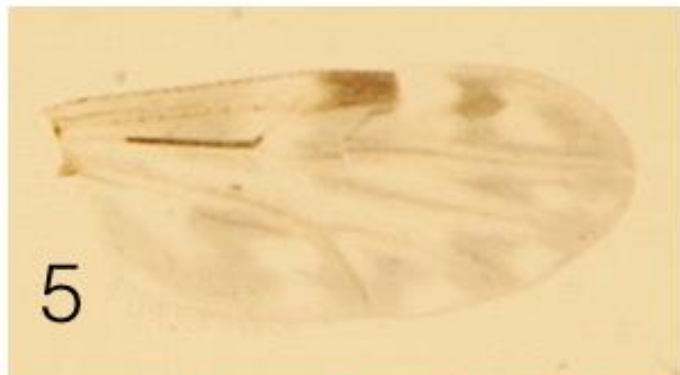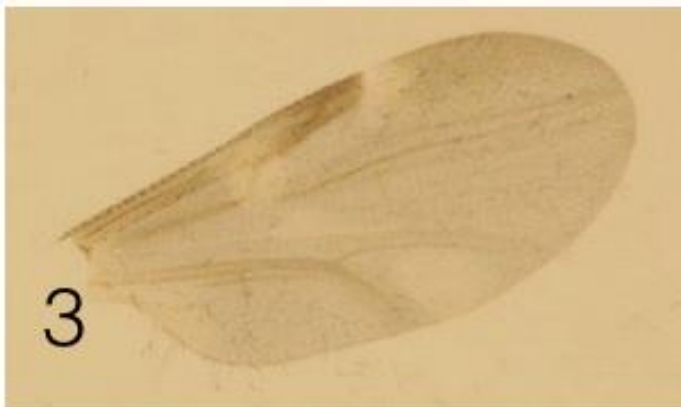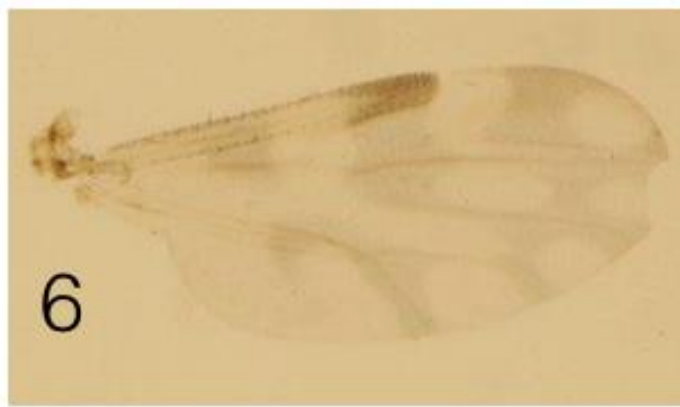

1. *C. punctatus*
2. *C. punctatus* P
3. *C. kibunensis*

4. *C. puncticollis* (unfed)
5. *C. riethi*
6. *C. griseidorsum*

Supplement: Supplementary file 3 — Additional file 3: Figure S1. Wing patterns for C. punctatus, C. punctatus P, C. kibunensis, C. puncticollis, C. riethi and C. griseidorsum collected in this study. [file 13071_2020_3938_MOESM3_ESM.pdf]
